# Vitamin D and Its Target Genes

**DOI:** 10.3390/nu14071354

**Published:** 2022-03-24

**Authors:** Carsten Carlberg

**Affiliations:** 1 Institute of Animal Reproduction and Food Research, Polish Academy of Sciences, PL-10-748 Olsztyn, Poland; c.carlberg@pan.olsztyn.pl; 2 Institute of Biomedicine, School of Medicine, University of Eastern Finland, FI-70211 Kuopio, Finland

**Keywords:** vitamin D, VDR, target genes, chromatin, epigenome, transcriptome, vitamin D signaling

## Abstract

The vitamin D metabolite 1α,25-dihydroxyvitamin D_3_ is the natural, high-affinity ligand of the transcription factor vitamin D receptor (VDR). In many tissues and cell types, VDR binds in a ligand-dependent fashion to thousands of genomic loci and modulates, via local chromatin changes, the expression of hundreds of primary target genes. Thus, the epigenome and transcriptome of VDR-expressing cells is directly affected by vitamin D. Vitamin D target genes encode for proteins with a large variety of physiological functions, ranging from the control of calcium homeostasis, innate and adaptive immunity, to cellular differentiation. This review will discuss VDR’s binding to genomic DNA, as well as its genome-wide locations and interaction with partner proteins, in the context of chromatin. This information will be integrated into a model of vitamin D signaling, explaining the regulation of vitamin D target genes.

## 1. Introduction

For 100 years, the term “vitamin D” has been used [1] for a molecule, the deficiency of which can lead to bone malformations, such as rickets [2]. More than 50 years ago, evidence accumulated that vitamin D_3_ acts via its metabolites 25-hydroxvitamin D_3_ (25(OH)D_3_) and, in particular, via the nuclear hormone 1α,25-dihydroxyvitamin D_3_ (1,25(OH)_2_D_3_) [3]. The emerging endocrinology of vitamin D was completed through the identification of vitamin D-binding proteins [4,5] and the cloning of VDR in different species [6,7]. VDR turned out to be an endocrine member of the nuclear receptor superfamily [8,9], suggesting that (similar to the steroid hormones estradiol, testosterone, progesterone, cortisol and mineralocorticoids, the vitamin A derivative all-*trans* retinoic acid and the thyroid hormone triiodothyronine) 1,25(OH)_2_D_3_ acts at nanomolar, or even picomolar, concentrations, as a direct regulator of specific target genes [10,11,12], in VDR-expressing tissues and cell types (www.proteinatlas.org/ENSG00000111424-VDR/tissue, accessed on 5 March 2022). The nearly ubiquitous expression of the VDR gene supports findings obtained during the past 30 years, that vitamin D regulates not only calcium homeostasis [13], but also immunity, cell growth and differentiation, as well as energy metabolism [14,15,16].

The genomic actions of vitamin D depend on the activation of VDR by 1,25(OH)_2_D_3_ and involve changes in the epigenome, leading to changes in the transcriptome and proteome. Therefore, in a typical in vitro vitamin D stimulation experiment, where supra-physiological concentrations of 10–100 nM 1,25(OH)_2_D_3_ are applied to a cell culture model, it takes (due to the time needed for RNA and protein synthesis) a few hours before the physiological effects of the nuclear hormone can be observed [17]. This is in contrast to the so-called non-genomic actions of vitamin D that happen within seconds to minutes and do not to involve VDR and changes in gene expression [18,19]. However, the timing of vitamin D signaling may not be of critical importance, since the physiology of vitamin D and its metabolites aims towards homeostasis, i.e., in vivo, there are no larger fluctuations in the concentrations of 1,25(OH)_2_D_3_ [20]. Thus, in net effect, the physiology of 1,25(OH)_2_D_3_ largely overlaps with the actions of the transcription factor VDR.

This review will outline VDR’s DNA-binding modes, genome-wide locations, as well as its interaction with chromatin components and other partner proteins. This will provide the basis of a model of vitamin D signaling that explains the mode of action of vitamin D target genes and allows for their classification.

## 2. VDR: A Transcription Factor Activated by Vitamin D

Transcription factors are proteins that are able to bind sequence-specifically to genomic DNA and interact with other nuclear proteins, which modulates the activity of RNA polymerase II and mRNA production [21,22]. Some of the approximately 1600 transcription factors encoded by the human genome are constitutively active and regulated primarily by their expression, while most of them are activated by extra- and intracellular signals. A few of these signal-dependent transcription factors are located either in a latent form in the cytosol and activated through translocation into the nucleus, or—most of them—are found in the nucleus and modulated in their activity by post-translational modifications, such as phosphorylation or acetylation. Furthermore, some members of the nuclear receptor superfamily have an additional mechanism of activation, which is a ligand-induced conformational change on the surface of their ligand-binding domain (LBD) [23,24].

**Figure 1:**
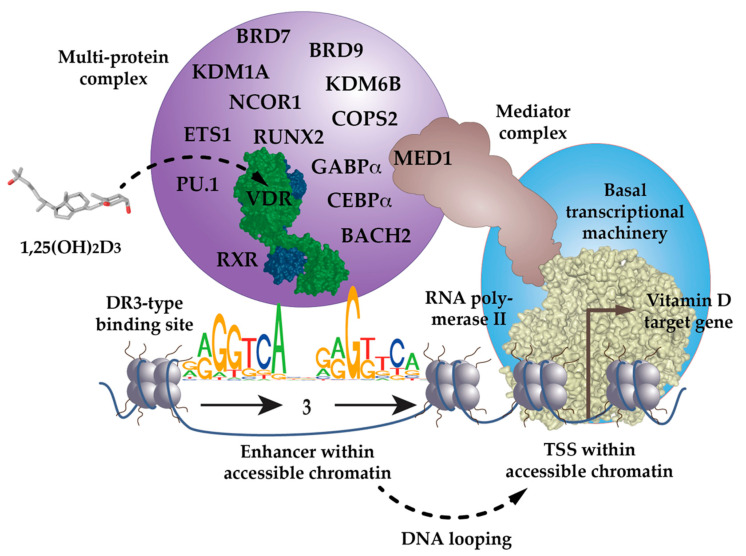
VDR as the key ligand-inducible component of a multi-protein complex. VDR is a part of a multi-protein complex that, e.g., contains co-receptors (RXR), pioneer factors (PU.1, CEBPα, GABPα, ETS1, RUNX2, BACH2), chromatin modifiers (KDM1A, KDM6B), chromatin remodelers (BRD7, BRD9), co-activators (MED1) and co-repressors (NCOR1, COPS2). The complex is activated through the binding of 1,25(OH)_2_D_3_ to VDR and attaches preferentially to DR3-type binding sites within enhancer regions. The mediator complex connects the activated VDR complex with the RNA polymerase II waiting on transcription start site (TSS) regions of vitamin D target genes. In most cases the linear distance of enhancer and TSS region are multiple kb, so that the intervening genomic DNA forms a regulatory loop. In this way the expression of the vitamin D target genes is either increased or decreased.

The inner surface of VDR’s LBD forms a ligand-binding pocket, where 40, mostly non-polar, amino acids snugly enclose the molecule 1,25(OH)_2_D_3_, so that it binds with an affinity of 0.1 nM [25]. This is a very high affinity, even in comparison with other nuclear receptors [26]. Ligand binding changes VDR’s interaction profile with many of the more than 50 nuclear proteins that have been reported to cooperate with the receptor [27]. Some of these VDR-interacting proteins function as co-repressors, such as NCOR1 (nuclear receptor corepressor 1) [28] or COPS2 (COP9 signalosome subunit 2, also called ALIEN) [29], co-activators of the NCOA (nuclear receptor coactivator) family [30], or members of the Mediator complex, such as MED1 [31,32,33] (Figure 1). Other VDR partner proteins are chromatin-modifying enzymes, such as histone acetyltransferases (HATs) [30], histone deacetylases (HDACs) [29], lysine demethylases, such as KDM6B [34] and KDM1A [35] or chromatin remodeling proteins, such as BRD (bromodomain-containing) 7 and 9 [36]. The large variety of its protein interaction partners suggests that VDR is a dynamic member of a large nuclear protein complex [37] (Section 5).

Many in vitro studies indicated that VDR binds efficiently to genomic DNA in a complex with the nuclear receptor retinoid X receptor (RXR) [38,39,40]. The preferred binding sites for the heterodimeric VDR-RXR complex are RGKTSA (R=A or G, K=G or T, S=C or G) sequence motifs, arranged as a direct repeat with three spacing nucleotides (DR3) [41,42,43] (Figure 1). However, these genomic binding sites need to be accessible to VDR complexes; i.e., they need to be located within open chromatin, which is referred to as euchromatin (Section 3). Chromatin immunoprecipitation sequencing (ChIP-seq) is a next-generation sequencing method that is able to determine, in an unbiased fashion, the genome-wide binding pattern, the so-called cistrome, of a transcription factor, such as VDR. ChIP-seq for VDR had been performed in many cellular models (Section 4), which confirmed that DR3-type binding sites are the most enriched sequence motifs below the summits (±100 bp) of VDR peaks [44]. However, depending on the threshold settings of DNA motif-finding algorithms, such as HOMER [45], only 10–20% of all VDR binding sites contain DR3-type motifs [44,46]. Thus, in a genome-wide perspective, not all VDR-containing nuclear complexes contact genomic DNA via DR3-type binding sites, by some distance.

The low percentage of DR3-binding VDR complexes detected by ChIP-seq also suggests that there are a number of scenarios in which VDR acts independently of RXR. VDR may use other nuclear proteins as alternative cooperative binding partners on genomic DNA [47,48,49] or may bind indirectly to DNA, such as “backpack”, to other transcription factors [50]. For example, below VDR ChIP-seq peak binding sites for the pioneer transcription factor PU.1 (purine-rich box-1) are enriched [44]. In fact, in THP-1 monocytic leukemia cells, the presence of PU.1 is observed on two-thirds of VDR’s genomic binding sites [51]. This makes sense, since PU.1, VDR and the pioneer factor CEBPα (CCAAT enhancer binding protein alpha) are the key transcription factors directing the differentiation of myeloid progenitor cells into monocytes and granulocytes, during the process of hematopoiesis [52]. Interestingly, THP-1 cells CEBPα [53], GABPα (GA-binding protein transcription factor alpha) [54] and ETS1 (ETS proto-oncogene 1, transcription factor) [55] also co-locate with VDR binding sites and act as pioneer factors for vitamin D signaling (Figure 1). Furthermore, in osteoblasts, vitamin D signaling is enhanced by the pioneer factors CEBPα and RUNX2 (RUNX family transcription factor 2) [56], while in T cells, this is mediated by the transcription factor BACH2 (BTB domain and CNC homolog 2) [57]. Thus, VDR uses help from RXR, but also from many other transcription factors, in order to form functional complexes with genomic DNA.

## 3. Vitamin D Target Gene Regulation in the Context of Chromatin

**Figure 2:**
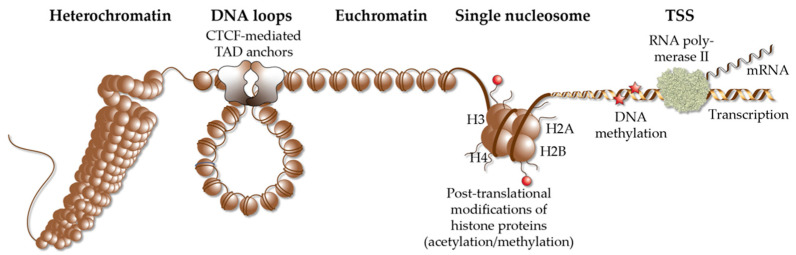
Elements of chromatin. Different elements of chromatin are shown, such as densely packed heterochromatin, DNA loops, such as TADs that are anchored by CTCF proteins, accessible euchromatin, the structure of a single nucleosome, chromatin modification via histone acetylation and methylation as well DNA methylation and a TSS, from which RNA polymerase II starts gene transcription into mRNA.

Each of the approximately 20,000 protein-coding genes of the human genome has one or multiple transcription start sites (TSSs). The latter are core promoter regions, to which RNA polymerase II is directed by general transcription factors, such as TBP (TATA box binding protein) [58]. Depending on a direct interaction of this so-called basal transcriptional machinery, with signal-dependent transcription factors (Section 2) or an indirect interaction via members of the Mediator complex [59], the RNA polymerase modulates the transcription rate of the respective gene, i.e., its expression (mRNA level) increases or decreases. Transcription factors, such as VDR, bind to enhancer regions [60], which are stretches of genomic DNA that contain specific binding sites (Section 2), for one or multiple transcription factors. The interaction of the protein complexes formed on TSS and enhancer regions is facilitated by DNA looping, in a so-called regulatory loop (Figure 1). Therefore, enhancers are equally likely found upstream and downstream of TSS regions [61]. However, both types of genomic regions need to be located within the same TAD (topologically associating domain), in order to efficiently interact. TADs are far larger loops of genomic DNA than regulatory loops, with a size of hundreds of kb to a few Mb [62]. They subdivide the human genome into at least 2000 units, which are functionally insulated from each other [63] (Figure 2). The borders of TADs are defined by the binding of the chromatin-organizing protein CTCF (CCCTC-binding factor) [64,65], forming together with cohesin and other proteins, so-called TAD anchors [66]. The interaction of the VDR-bound enhancers with genomic regions outside of a TAD are prevented by these insulating TAD borders. This is the reason why genes are regulated almost exclusively by enhancers that are located within the same TAD. This also implies that the linear distance between VDR-bound enhancers and TSS region(s) cannot be larger than the size of the respective TAD.

In a repeating unit of 200 bp, genomic DNA is packaged around nucleosomes, which are complexes of two copies of each of the histone proteins H2A, H2B, H3 and H4 (Figure 2). The complex of nucleosomes and genomic DNA is referred to as chromatin and can be interpreted as the physical expression of the epigenome [67]. Chromatin regions largely differ in their degree of packaging [68], so that transcription factors are limited in their access to genomic binding sites [69]. The accessibility of chromatin can be monitored genome-wide by the methods of DNase-seq (DNase I hypersensitivity sequencing) [70], FAIRE-seq (formaldehyde-assisted isolation of regulatory elements sequencing) [71] and ATAC-seq (assay for transposase-accessible chromatin using sequencing) [72]. The vast majority of the genome is covered in a cell- and tissue-specific fashion by densely packed heterochromatin, which is largely inaccessible, in order to prevent the unintentional activation of genes. In contrast, in an average differentiated cell, genomic DNA is accessible at less than 200,000 loci (representing only some 10% of the whole genome) that primarily comprise TSS and enhancer regions [61]. This has a key impact on vitamin D signaling, since VDR binds exclusively to accessible enhancer regions (Section 4) and activates only those genes, the TSS region of which are located within euchromatin.

The distribution of eu- and heterochromatin (including their specific epigenetic markers) of a given cell is referred to as its epigenetic landscape or epigenome [73]. The epigenome is determined by patterns of DNA methylation, post-translational modification of histone tails and 3-dimensional chromatin organization [74]. It depends on the activity of chromatin-modifying enzymes, such as DNA methyltransferases (DNMTs), which add methyl groups to cytosines within genomic DNA, TET (Tet methylcytosine dioxygenase) proteins that initiate DNA methylation, or HATs, HDACs, lysine methyltransferases (KTMs) and KDMs, which add or remove acetyl and methyl groups to histones [75]. Histone acetylation is generally associated with transcriptional activation, but it is not important which exact amino acid is acetylated. In contrast, for histone methylation, the exact residue and its degree of methylation (mono-, di- or tri-methylation) is critical. Furthermore, the function of chromatin-remodeling enzymes is to shift or evict nucleosomes, in an ATP-dependent fashion. The projects ENCODE (www.encodeproject.org, accessed on 5 March 2022) [61] and Roadmap Epigenomics (www.roadmapepigenomics.org, accessed on 5 March 2022) [76] systematically assessed the epigenomes of more than 100 human cell lines, as well as primary cells, and serve as a reference for the epigenome of non-stimulated human tissues and cell types. However, in contrast to the static genome, the epigenome dynamically responds to intra- and extracellular signals, since chromatin modifying enzymes are often the endpoints of transduction cascades of peptide hormones, cytokines and growth factors [77]. Thus, the response of the epigenome to signals, such as 1,25(OH)_2_D_3_, is even more important than its ground state.

Nuclear hormones, such as 1,25(OH)_2_D_3_, affect the epigenome via direct interaction of their receptors with chromatin-modifying enzymes (Section 2), as well as through up- or down-regulating the genes encoding for chromatin modifiers. In this way:

1. Vitamin D affects histone markers for active chromatin, such as H3K27ac (acetylated histone H3 at lysine 27), and for TSS regions, such as H3K4me3 (tri-methylated histone H3 at lysine 4) [53,57,78];
2. VDR initiates the demethylation of its binding sites via interaction with TET2 [79];
3. The accessibility of thousands of VDR-binding enhancer and TSS regions is affected by 1,25(OH)_2_D_3_ [80,81];
4. The binding of CTCF, to more than 1000 of its genomic sites, is modulated by 1,25(OH)_2_D_3_ [82];
5. The organization of some 400 TADs is dependent on 1,25(OH)_2_D_3_ [82], i.e., vitamin D affects the 3-dimensional chromatin structure.

Thus, there are multiple ways by which 1,25(OH)_2_D_3_ modulates the epigenome of its target tissues. Interestingly, some 1,25(OH)_2_D_3_-modulated chromatin loci, such as TSS regions, open only 2 h after ligand stimulation, while most sites take 24 h to reach maximal accessibility [78,80]. This suggests that many effects of vitamin D on the epigenome are secondary, i.e., they are mediated by genes and proteins that are primary vitamin D targets [83,84]. Nevertheless, the vitamin D-triggered effects on the epigenome facilitate the looping of VDR-bound enhancers, towards accessible TSS regions within the same TAD [85]. This assembly of enhancer and TSS regions enables the formation of a large protein complex, containing VDR, nuclear adaptor proteins, chromatin-modifying enzymes and RNA polymerase II, modulating gene transcription (Section 2) (Figure 1).

## 4. Genome-Wide Location of VDR

In human cellular systems, the VDR cistrome had been determined in B cells (GM10855 and GM10861) [86], T cells [87], macrophages (lipopolysaccharide-polarized THP-1) cells [44], peripheral blood dendritic cells [79], colorectal cancer cells (LS180) [88], prostate epithelial cells (RWPE1) [89], hepatic stellate cells (LX2) [90] and human kidney tissue [91]. However, the VDR cistrome was studied in most detail in monocytes (undifferentiated THP-1 cells) [46,92]. For comparison, the mouse VDR cistrome was obtained in pre-adipocytes (3T3-L1) [93], osteocytic cells (IDG-SW3) [94], pre-osteoblastic and differentiated osteoblastic cells (MC3T3-E1) [95], as well as in mouse intestine [96], mouse kidney [91] and bone-marrow-derived mesenchymal stem cells [56]. In the presence of 1,25(OH)_2_D_3_, the VDR cistrome comprises 5000–20,000 genomic loci, which represents a 2- to 10-fold increase compared to respective unstimulated cells. However, this implies that the VDR cistrome contains a lower number of persistent loci that remain constantly occupied, while their occupancy significantly changes after stimulation with a ligand [46]. These persistent VDR binding sites are the primary contact points of the human genome with 1,25(OH)_2_D_3_ and are considered as “hotspots” for vitamin D signaling (Section 6). These sites coordinate the functional consequences of ligand stimulation over time, i.e., these sites best represent the spatio-temporal response of the (epi)genome to the extracellular changes in vitamin D levels. In addition, there are transient VDR-binding loci that modulate the response of the epigenome to vitamin D and support persistent VDR sites. Thus, the genome-wide, ligand-induced binding of VDR, to its preferred loci, is the most prominent of all the epigenome-wide effects of vitamin D.

## 5. Model of Vitamin D Signaling

The here-presented model of vitamin D signaling describes the sequential activation of vitamin D target genes [97]. Typically, protein-coding target genes of vitamin D are considered, but there are also some non-coding RNA genes that are known to be modulated by vitamin D [98,99,100]. VDR does not act as an isolated protein but functions in the context of a larger, dynamically composed protein complex that contains RXR, other possible co-receptors, pioneer factors, such as PU.1, CEBPα, GABPα, ETS1, RUNX2 and BACH2, co-factors, chromatin modifiers and chromatin remodelers (Figure 1).

1. The pioneer factors within the complex may take the first contact to enhancer regions. With the help of chromatin-remodeling proteins, they optimize the access of VDR to suitable binding motifs within the enhancer region, including the demethylation of genomic DNA.
2. Chromatin modifiers within the complex then leave marks, such as H3K27ac, to the local chromatin region.
3. Although VDR may not be the first protein of the complex making contact with the enhancer region, its specific activation by 1,25(OH)_2_D_3_ drives the activity of the other members of the complex. This may explain the epigenetic effects of 1,25(OH)_2_D_3_, such as chromatin opening, histone marks and the recruitment of pioneer factors.
4. When the complex is established on the enhancer region, DNA looping events to TSS regions within the same TAD region, which are complexed with a basal transcriptional machinery, become stabilized. Via 1,25(OH)_2_D_3_-triggered effects on CTCF-dependent TAD anchor formation, this also affects the structure of the whole TAD.

In terms of net effect, the epigenome-modulating functions of vitamin D will increase or decrease the activity of RNA polymerase II, so that the mRNA expression of the respective gene(s) changes. On persistent VDR binding sites, i.e., enhancer with residual VDR binding, even in the absence of a ligand, the above-described multi-step process happens more efficiently, explaining why these loci are the primary sites of 1,25(OH)_2_D_3_-dependent gene expression.

## 6. Vitamin D Target Genes

Vitamin D target genes are detected via a statistically significant change in their expression, within a given time frame (often 24 h), after ligand stimulation. Long-time known vitamin D target genes, such as *BGLAP* (bone gamma-carboxyglutamate protein, also called osteocalcin) [101] or *CAMP* (cathelicidin antimicrobial peptide) [102], were deduced based on the observation of the physiological effects of vitamin D, e.g., on calcium homeostasis or the defense against microbe infection, respectively. On the level of mRNA changes, vitamin D target genes were analyzed, initially, by single gene approaches, using northern blotting or quantitative PCR. After the completion of the human genome, microarrays became popular, which are able to detect the transcriptome-wide effects of vitamin D stimulation in one assay [103,104]. However, for some 10 years, RNA sequencing (RNA-seq) has been the method of choice for describing the vitamin D-dependent transcriptome [105]. Studies in a large number of cellular models led to a tremendous increase in the number of putative vitamin D target genes. For example, a microarray in THP-1 cells reported, in one experimental setting, 3372 significantly (false discovery rate (FDR) < 0.05) regulated genes [92], while in another microarray, in the same cellular model, 4532 genes passed the statistical threshold [106]. Interestingly, 1227 genes were common and more than half of them (695) were confirmed by RNA-seq [46,80]. Thus, in a given cellular model, it is more likely that a few hundred genes respond to vitamin D than thousands of gene candidates. Interestingly, a meta-analysis of transcriptome-wide investigations of vitamin D target genes in 94 different human and mouse cell models resulted in only two common targets, *CYP24A1* (cytochrome P450 family 24 subfamily A member 1) and *CLMN* (calmin) [107]. Thus, vitamin D target genes are largely tissue specific.

**Figure 3:**
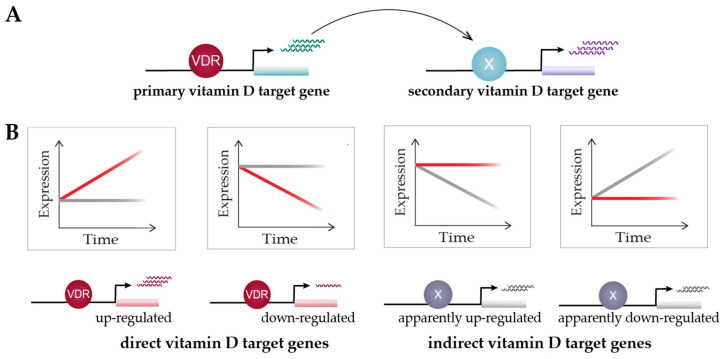
Classification of vitamin D target genes. Primary vitamin D target genes are directly regulated by VDR, while secondary vitamin D targets are controlled by transcriptional regulators that are encoded by primary targets (**A**). Time course analysis allows to differentiate vitamin D target genes in four different types based on cause and direction of expression change [108] (**B**). This suggests an alternative view on vitamin D signaling: 1,25(OH)_2_D_3_ either directly induces or reduces the expression of its target genes via VDR or prevents their expression change mediated by other factors. Red and grey lines indicate gene’s expression level in the presence or absence of 1,25(OH)_2_D_3_, respectively.

Time course analysis of vitamin D target genes allows one to classify them into rapidly responding (4–8 h) “primary” target genes and delayed-reacting “secondary” targets (Figure 3A). Primary target genes are directly regulated by 1,25(OH)_2_D_3_-activated VDR, as described in the model of vitamin D signaling (Section 5); i.e., these genes need to have, within the same TAD, a VDR-binding enhancer. In contrast, secondary target genes may be regulated by transcription factors, co-factors or chromatin modifiers, which are encoded by primary vitamin D target genes (Figure 3A). Suitable proteins encoded by primary target genes are the transcription factors BCL6 (B-cell CLL/lymphoma 6), NFE2 (nuclear factor, erythroid 2), POU4F2 (POU class 4 homeobox 2) and ELF4 (E74-like factor 4) in THP-1 cells [83], as well as IRF5 (interferon regulatory factor 5), MAFF (MAF BZIP transcription factor F), MYCL (MYCL proto-oncogene, BHLH transcription factor), NFXL1 (nuclear transcription factor, X-box binding-like 1) and TFEC (transcription factor EC), as well as the transcriptional co-regulators MAMLD1 (mastermind-like domain-containing 1), PPARGC1B (PPARG coactivator 1 beta), SRA1 (steroid receptor RNA activator 1) and ZBTB46 (zinc finger and BTB domain-containing 46) in human PBMCs (peripheral blood mononuclear cells) [108]. In this way, secondary target genes do not have to carry a VDR-binding enhancer within their TADs. For example, although the time course study in human PBMCs used a strict statistical approach (threshold testing applying a fold change (FC) > 1.5 and 2), 662 vitamin D-responding genes were identified (FDR < 0.05), 179 of which are primary and 483 secondary targets [108]. An alternative classification of the same set of genes suggests that 293 of them are direct and 369 indirect targets of vitamin D (Figure 3B). Irrespective of the timing of their response to 1,25(OH)_2_D_3_, the expression change of direct targets is driven by VDR-bound enhancers, while indirect targets are primarily stabilized in their expression by 1,25(OH)_2_D_3_ and its receptor, against an up- or down-regulation by other transcription factors and/or their epigenetic effects.

The model of vitamin D signaling (Section 5) illustrates the mechanisms of up-regulation of primary vitamin D target genes. However, the majority of vitamin D target genes are down-regulated, in particular, when cells are stimulated with 1,25(OH)_2_D_3_ for 24 h or longer. The down-regulation of a gene by vitamin D is only possible when the gene is first up-regulated by other transcription factors or epigenetic effects, mediated by chromatin modifiers. The most, likely mechanism of down-regulation of a gene is to block one or several of its up-regulating factors. Accordingly, the majority of down-regulated target genes could be classified as indirect targets, i.e., vitamin D counteracts their up-regulation rather than prominently down-regulating their expression [108]. For example, VDR antagonizes pro-inflammatory transcription factors, such as NFAT, AP1 and NFκB, in immune cells [109]. However, since each gene is up-regulated by an individual set of transcription factors and chromatin modifiers, there are also individual mechanisms of its down-regulation. Thus, there is no general model for describing the mechanism of the down-regulation of vitamin D target genes.

The expression of the majority of vitamin D target genes is less than 5-fold, up- or down-regulated (after a stimulation for 24 h with 1,25(OH)_2_D_3_); i.e., only a few genes respond with huge expression changes to vitamin D. For example, in THP-1 cells, the top five are the up-regulated genes *CYP24A1*, *CAMP*, *TSPAN18* (tetraspanin 18), *CD14* (CD14 molecule) and *FBP1* (fructose-bisphosphatase 1), with an FC of expression ranging from 47 to 402 [80]. In human PBMCs, *CYP24A1* and *CAMP* also have an FC of 409 and 158, respectively, the top up-regulated vitamin D target genes, but *STEAP4* (STEAP4 metalloreductase), *NRG1* (neuregulin 1) and *CXCL10* (C-X-C motif chemokine ligand 10) show an FC of 471, 450 and 158, respectively, meaning comparable levels of down-regulation of their expression [108]. This prominent down-regulation of expression is more remarkable than the up-regulation, since it is far easier to increase a very low basal expression than a down-regulation of a highly expressed gene. Nevertheless, one should remember that these in vitro 1,25(OH)_2_D_3_ experiments are designed for maximal effects and do not reflect the reality of the endocrinology of vitamin D in vivo [110,111].

## 7. Functional Profile of Vitamin D Target Genes

The most important thing to consider in the analysis of lists of hundreds of vitamin D target genes is the identification of the underlying biological processes. This is often evaluated by gene ontology analysis, where the list of target genes is assessed for statistically significant enrichment of a predefined list of terms, concerning (i) the molecular function, i.e., the molecular activity of a gene, (ii) the biological process, i.e., the cellular or physiological role carried out by a gene in the context of other genes, and (iii) the cellular component, i.e., the location where the gene’s product functions in the cell. For example, in THP-1 cells, the biological processes “neutrophil activation”, “inflammatory response”, “neutrophil degranulation”, “negative regulation of T cell proliferation” and “positive regulation of cytokine secretion”, are most significantly associated with the list of 695 vitamin D target genes [84]. This fits with the known key functions of vitamin D in monocytes [112]. Since monocytes are the most vitamin D responsive cell fraction of PBMCs, gene ontology analysis of the 662 vitamin D target genes in this primary tissue suggests similar functions, such as neutrophil degranulation”, “inflammatory response”, “cytokine-mediated signaling pathway”, “extracellular matrix organization” and “positive regulation of angiogenesis” [108]. For example, vitamin D down-regulates 10 of 12 *HLA* (human leukocyte antigen) class II genes and five *S100A* (S100 calcium-binding protein A) genes encoding for alarmins, as well as modulating the expression of six members of the *CXCL* gene family, encoding for chemokines [108]. The resulting vitamin D-triggered immune tolerance leads to the induction of regulatory T cells, which down-regulate the activity of other cells in the immune system [57,79]. This is the central mechanism for how vitamin D dampens chronic inflammation and autoimmunity in diseases, such as inflammatory bowel disease [113] and multiple sclerosis [114].

VDR is expressed in many cell types (Section 1), but most of them differ majorly in their respective epigenetic landscape and, thus, expression of vitamin D target genes. Therefore, there are a large variety of biological processes being regulated by 1,25(OH)_2_D_3_, i.e., in summary of all VDR-expressing tissues, vitamin D has rather ubiquitous functions.

## 8. Conclusions and Future View

Although vitamin D was discovered through its critical role in calcium homeostasis, being essential for proper bone formation, vitamin D signaling is studied most intensively, nowadays, in the immune system [115]. Therefore, the present molecular understanding of vitamin D signaling (Section 5) is mainly based on the integration of data obtained in immune cells [97].

The main challenge for future investigations of vitamin D target genes is their analysis in a human in vivo setting. The first studies involving a transcriptome- and epigenome-wide analysis, performed directly after isolation of PBMCs from vitamin D_3_-supplemented human donors, have already begun [116,117,118]. These, and similar types of investigations, may provide us with a further enhanced understanding of the action of VDR and its target genes, in particular, in the context of immunity.
